# Pollination Under Pressure: Grazing and Nutrient Enrichment Reshape Arctic Pollinator Communities and Interaction Networks

**DOI:** 10.1111/gcb.71039

**Published:** 2026-08-08

**Authors:** Nicolina Johanson, Martin Andrzejak, Harry Olde Venterink, Luísa G. Carvalheiro, Anu Eskelinen, Risto Virtanen

**Affiliations:** ^1^ Ecology and Genetics University of Oulu Oulu Finland; ^2^ Research Group WILD Vrije Universiteit Brussel Brussel Belgium; ^3^ Ecology Department Universidade Federal de Goiás Goiânia Brazil; ^4^ German Centre for Integrative Biodiversity Research iDiv Leipzig Germany

**Keywords:** Arctic, diversity, grazing, land‐use change, nutrient enrichment, pollinator networks

## Abstract

Pollinators are declining globally due to anthropogenic pressures, such as intensive grazing and nutrient enrichment. Yet, their combined effects on pollinators and plant‐pollinator interactions remain poorly understood, particularly in the Arctic. Here, we experimentally tested how grazing exclusion and nutrient addition shape flower‐visiting insect communities and interaction networks in a high‐productivity montane and a low‐productivity tundra grassland, in northern Fennoscandia. Grazing exclusion emerged as the primary driver across both grasslands, increasing flower‐visitor family richness, diversity, and the number of plant‐pollinator interactions. These effects were especially strong in the tundra grassland, where resource limitation is more pronounced. In contrast, nutrient addition had weaker and more context‐dependent effects, enhancing flower‐visitor richness and the number of interactions mainly in the tundra grassland and particularly when combined with grazing exclusion. At both sites, grazing exclusion and nutrient addition altered community composition, with the strongest shifts occurring under their combined effects. Despite large effects on interaction numbers, network structure responded more subtly. Weighted connectance declined with grazing exclusion, especially in the tundra, indicating that interactions became more concentrated on a subset of plant species where floral resources were abundant. In contrast, network specialization (H_2_’) and nestedness showed limited responses to experimental treatments, suggesting relatively stable interaction organization. Overall, our results demonstrate that grazing strongly regulates pollinator communities and interaction networks in Arctic grasslands, while nutrient enrichment plays a secondary, context‐dependent role. The pronounced sensitivity of tundra grasslands highlights their vulnerability to environmental change and underscores the importance of managing grazing pressure to sustain pollination processes in Arctic ecosystems.

## Graphical abstract

Grazing exclusion reshaped Arctic flower‐visitor communities, increased plant‐pollinator interactions, and altered interaction networks, while nutrient enrichment had weaker, context‐dependent effects. Together the two drivers produced both independent and interactive effects, with tundra grasslands showing the greatest sensitivity to environmental changes.

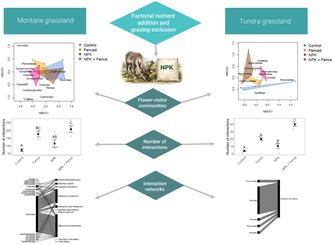

## 1 Introduction

Pollinating insects are declining globally at an unprecedented rate because of land‐use intensification, habitat loss, climate change, and other anthropogenic pressures (Biesmeijer et al. 2006; Baldock et al. 2013; IPBES 2016; Dicks et al. 2021; Millard et al. 2021). Beyond altering abundance, diversity and community composition of species, these drivers can restructure plant‐pollinator interaction networks, with consequences for ecosystem functioning, stability and resilience (Tylianakis et al. 2010; Vanbergen and Insect Pollinators Initiative 2013). Environmental disturbances often shift communities toward dominance by a few generalist species, simplifying interaction networks and potentially increasing their vulnerability to further environmental change (Aizen and Feinsinger 1994; Spiesman and Inouye 2013). Because pollinator declines are linked to losses of floral resources or microhabitats required for nesting or larval development (Potts et al. 2005; Klein et al. 2007), understanding how land‐use drivers alter plant‐pollinator interactions through resource availability is essential for predicting the future stability of pollination services under global change.

In grassland ecosystems, grazing regimes and nutrient inputs are among the most pervasive land‐use drivers shaping plant‐pollinator systems (Potts et al. 2010; Dicks et al. 2021). These processes primarily influence pollinator communities indirectly, by modifying plant community composition, vegetation structure, and floral resource availability (Burns et al. 2022; Johanson et al. 2025). Floral resource quantity and composition can be seen as predictors of pollinator abundance and foraging behavior (Roulston and Goodell 2011; Lázaro et al. 2016), and reductions in flowering plants can disrupt plant‐pollinator interactions and network stability (Winfree et al. 2009).

Of these drivers, nutrient enrichment influences plant‐pollinator interactions mainly through bottom‐up effects on plant and flower communities (Burkle et al. 2013; David et al. 2019). In nutrient‐limited systems, increases in nutrient availability enhance primary productivity and floral display by increasing flower size and abundance, potentially attracting more flower‐visitors (Galen 1985; Schemske and Horvitz 1988; Makino et al. 2007). At the same time, nutrient enrichment often reduces flowering plant diversity by promoting competitive dominance of fast‐growing species, which are often wind‐pollinated such as graminoids (Campbell and Halama 1993; Wyka and Galen 2000; Wang et al. 2022; Nelson et al. 2025; Johanson et al. 2025). Nutrient enrichment can also modify pollen and nectar quality, with potential consequences for pollinator nutrition and foraging behavior (Vaudo et al. 2016; Carvalheiro et al. 2020; Carvalheiro et al. 2023). Consequently, while some plant species may experience higher visitation rates, overall interaction diversity often declines as pollinators concentrate on the most abundant or rewarding plant species, reducing per‐flower visitation and increasing pollen limitation (Munoz et al. 2005; Tadey 2015). Pollinator taxa often respond unevenly to flower resources; specialized pollinators may decline with the loss of preferred resources, whereas highly generalized taxa may persist or increase, leading to greater niche overlap, thereby altering network structure (Wang et al. 2022).

Large‐herbivore grazing is a widespread land‐use driver that can profoundly restructure plant‐pollinator systems, often with far‐reaching ecological consequences (Rakosy et al. 2022). Through biomass removal, trampling, and nutrient redistribution grazers influence plant community structure, flowering phenology and the availability of floral and nesting resources for pollinators (Vázquez and Simberloff 2004; Potts et al. 2005; Pykälä 2005; Vanbergen and Insect Pollinators Initiative 2013). Although some plant species compensate for herbivory by increasing flower production, thereby attracting more pollinators, such responses depend on herbivore identity, grazing intensity and habitat type (Moeller and Geber 2005; Robinson et al. 2018). While intensive grazing substantially reduces aboveground vegetation, depletes floral resources, degrades insect habitats and nesting sites, and drives declines in flower‐visitor assemblages (Kruess and Tscharntke 2002; Hartley and Jones 2003; Filazzola et al. 2020; Rakosy et al. 2022), low‐ to moderate grazing can maintain open vegetation, prevent plant dominance, and promote spatial heterogeneity, potentially supporting more diverse and functionally varied pollinator communities (Lázaro et al. 2016; Rakosy et al. 2022).

Recent research suggests that grazing and nutrient enrichment can interact to affect plant‐pollinator interactions. Grazing can mitigate nutrient‐driven plant dominance by maintaining open patches and floral diversity, whereas nutrient enrichment under grazing exclusion can increase flower abundance while reducing community evenness (Veen et al. 2024; Johanson et al. 2025). Veen et al. (2024) found that nutrient addition reduced plant diversity, forb cover, and pollinator visitation, while grazing maintained or enhanced diversity. However, how these community‐level responses translate into changes in pollinator assemblages and network structure across ecosystems with contrasting productivity remains unclear. Moreover, most experimental studies have focused on single drivers in isolation, yet ecosystems are simultaneously exposed to multiple interacting global change factors. Because these factors can often be non‐additive and produce unexpected and emergent ecosystem responses, multifactor experimental approaches are needed to predict ecological dynamics under global change (Knorr et al. 2024).

Ecological network analyses provide a useful framework to address these gaps, as they capture structural properties that are not evident from species abundance or richness alone. Metrics such as network size (number of interactions), connectance, specialization (H_2_′) and nestedness describe complementary dimensions of interaction structure and potential robustness in mutualistic systems (Bascompte et al. 2003; Thébault and Fontaine 2010; Ramos‐Jiliberto et al. 2012; Landi et al. 2018). Grazing has been associated with alterations in network properties, often increasing network size, connectance and H_2_′ while reducing nestedness under high pressure, which can increase the vulnerability of specialist species and simplify networks (Bascompte and Jordano 2007; Rakosy et al. 2022). In contrast, plant‐pollinator networks appear relatively resistant to single‐nutrient additions, whereas combined nutrient enrichment can strongly restructure taxonomic composition and interaction patterns (Burkle and Irwin 2009; Wang et al. 2022).

Most empirical evidence on plant‐pollinator networks comes from temperate regions of North America and Europe, while grasslands in the Arctic remain underrepresented despite their high sensitivity to environmental change (Burns et al. 2022). Arctic grasslands present a particularly interesting context for studying plant‐pollinator dynamics. These ecosystems are characterized by harsh abiotic conditions, including short growing seasons, low nutrient availability, fast species turnover, and relatively low species richness (Havström et al. 1993; Körner 1999; Munoz et al. 2005; Arroyo et al. 2006), yet plant‐pollinator networks in these areas can exhibit surprising complexity (Olesen et al. 2008; Tiusanen et al. 2016). Arctic pollinator assemblages are often dominated by muscid flies, while hoverflies and bumblebees can be important contributors to pollination services (Zoller et al. 2023). Plant‐pollinator networks in these ecosystems are typically characterized by high generalism, with obligate specialist plants and pollinators being rare (Olesen and Jordano 2002). This may confer resilience to species loss, but it may also render these networks vulnerable to environmental change. Nevertheless, experimental evidence assessing the fragility of Arctic plant‐pollinator networks under environmental changes is very limited (Burns et al. 2022).

Here, we investigate how nutrient addition and grazing exclusion influence flower‐visiting insect communities and plant‐pollinator interaction structure in Arctic grasslands. We quantify treatment effects on flower‐visitor abundance, richness, and community composition and assess corresponding responses in network properties (network size, weighted connectance, specialization (H_2_’) and nestedness). By integrating community‐level responses with network structure, we aim to better understand the mechanisms by which nutrient availability and grazing regulate plant‐pollinator systems in Arctic ecosystems.

We hypothesize that (1) grazing exclusion and (2) nutrient addition alter flower‐visitor richness and diversity through their effects on floral resource availability. We expect grazing exclusion to increase visitor richness and diversity by allowing floral resources to accumulate in the absence of herbivory. Nutrient addition (N, P, K) is expected to increase visitor richness and diversity where it enhances flower abundance but decreases it where nutrient‐driven competition reduces flower density. We further expect the combined grazing exclusion and nutrient addition to produce the strongest positive effects on flower‐visitors as nutrient input can amplify the effects of grazing exclusion (Johanson et al. 2025). We further hypothesize that (3) grazing exclusion and nutrient addition reshape flower‐visitor assemblage composition in response to changes in floral community structure (Johanson et al. 2025). Finally, we predict that (4) these changes will scale up to affect interaction networks. Specifically, we expect network size (number of species and interactions) to increase under treatments that enhance floral resources, connectance to decline as increased flower‐visitor richness and the number of interactions outpace the proportion of realized links, and specialization (H_2_′) and nestedness to increase under conditions of high flower abundance that allow specialist visitors to interact with subsets of generalist plant species. We also expect the strength and direction of these responses to differ between sites due to variation in species composition and environmental conditions.

## 2 Methods

This study was conducted at two experimental grassland sites within the Nutrient Network (NutNet), a global research collaboration that investigates how nutrient availability and herbivory regulate grassland productivity and biodiversity using standardized experimental designs (Borer et al. 2014). The experimental sites were established in 2013–2014 in north‐western Finnish Lapland, near Lake Kilpisjärvi (69.05° N, 20.87° E). The region has a short growing season (June–September), a mean July temperature of 10°C, a mean annual temperature of −2°C, and a mean annual precipitation of 518 mm (Finnish Meteorological Institute, 1990–2020).

One site (kilp.fi, 730 m a.s.l.) is located above the treeline in a low‐productivity tundra grassland, while the second site (saana.fi, 600 m a.s.l.) lies below the treeline in a productive montane grassland. The tundra grassland consists of graminoid‐ and forb‐dominated meadow vegetation with species such as *Agrostis mertensii* , *Anthoxanthum nipponicum* , *Carex bigelowii* , *Carex lachenalii* , and *Festuca ovina* , interspersed with low‐growing forbs such as *Antennaria dioica* , *Sibbaldia procumbens* , *Solidago virgaurea*, and *Veronica alpina* , and creeping dwarf shrubs such as *Harrimanella hypnoides* and *Salix herbacea* . In this site, the mean vascular plant biomass of grazing exclosures was 113.1 g m^−2^ (SD 54.9, *n* = 4) in 2022 (R. Virtanen, A. Eskelinen, unpublished data, see also Yan et al. 2026). The montane grassland site is dominated by tall‐statured forbs such as *Anthriscus sylvestris, Geranium sylvaticum*, *Ranunculus subborealis*, and *Trollius europaeus* , along with legumes such as *Astragalus frigidus* , and graminoids such as *Anthoxantum nipponicum, Poa alpigena*, and *Poa alpina* . In this site, the mean vascular plant biomass of grazing exclosures was 250.2 g m^−2^ (SD 85.2, *n* = 4) in 2022 (R. Virtanen, A. Eskelinen, unpublished data, see also Yan et al. 2026).

Both sites are used as summer grazing rangelands for semi‐domesticated reindeer ( *Rangifer tarandus tarandus* ), which graze in the area from snowmelt in late May–June until August–September. Grazing intensity is highest shortly after snowmelt, when reindeer frequently consume developing flower stems, strongly reducing flower abundance (Johanson et al. 2025). Reindeer also reduce aboveground biomass later in the growing season, with reported biomass losses of up to 60% (Yan et al. 2026). Reindeer are generalist herbivores whose natural diet comprises a wide range of plant species (over 250 recorded), as well as lichens forming a major component, alongside grasses, herbs, and woody plants (Nieminen and Heiskari 1989). Other herbivores including Norway lemmings ( *Lemmus lemmus* ), grey‐sided voles ( *Myodes rufocanus* ), and mountain hares ( *Lepus timidus* ) occur at the study sites, but contribute minimally to herbivory on herbaceous vegetation.

At both sites we established a field experiment in 5 × 5 m plots arranged in a randomized block design following the standard NutNet protocol (Borer et al. 2014). The design included four blocks per site, each containing four plots (16 plots per site). The four plots were randomly assigned to the following treatments: Control, nutrient addition (NPK), grazing exclusion (Fence) and the combined treatment (NPK + Fence). Following the NutNet protocol (Borer et al. 2014), the NPK treatment corresponded to the full nutrient addition used in the multiple‐nutrient experiment and consisted of annual additions of nitrogen (N; 10 g m^−2^ year^−1^), phosphorus (P; 10 g m^−2^ year^−1^), and potassium (K; 10 g m^−2^ year^−1^), applied as granular urea (N), triple superphosphate (P), and potassium sulfate (K). Potassium addition also included a one‐time application of micronutrients in 2014 at the tundra site and in 2015 at the montane site: boron (B; 0.1 g m^−2^), calcium (Ca; 6 g m^−2^), copper (Cu; 1 g m^−2^), iron (Fe; 17 g m^−2^), magnesium (Mg; 3 g m^−2^), manganese (Mn; 2.5 g m^−2^), molybdenum (Mo; 0.05 g m^−2^), sulphur (S; 12 g m^−2^), and zinc (Zn; 1 g m^−2^). The grazing‐exclusion × nutrient‐addition experiment included two crossed treatments: nutrient addition (Control vs. NPK), and grazing exclusion (open vs. Fenced). Grazing exclusion was implemented using 110‐cm‐high fences, with the lower 80–90 cm covered by 1‐cm mesh and visible 10 mm‐wide polytape wires above. Fertilization and fencing began in 2014 at the tundra site and in 2015 at the montane site.

We conducted the flower visitation surveys during peak flowering in 2022 (2 July–30 July) to quantify plant‐pollinator interactions. Flower‐visiting insects were sampled from plots receiving the following treatments: Control, Fence, NPK and NPK + Fence (fenced NPK). We conducted the surveys under sunny to partly cloudy conditions with low wind. Each plot was surveyed twice daily: in the morning between 08:00 and 11:00 a.m., and in the afternoon between 13:00 and 16:00 p.m., with 20‐min observations, over 10 days. Sampling effort was standardized across plots, treatments and sites, and all surveys were conducted by a single observer. During observations, we walked slowly around each plot to minimize disturbance and maximize detection of flower‐visitors. We defined a visitation event as an insect contacting the reproductive parts of a flower (anthers or stigma); insects resting on petals were not recorded. Flower‐visiting insects were collected with a hand net and preserved in 90% ethanol unless they could be reliably identified in the field or documented photographically (e.g., Lepidoptera). We recorded the plant species visited for each interaction. All specimens were identified to family level with assistance from taxonomic experts at the Finnish Museum of Natural History (LUOMUS). Family‐level identification was considered appropriate because the focus of this study is on treatment effects on interaction structure, rather than species‐specific responses. In Arctic systems, pollinator communities are relatively species‐poor, and insect families often correspond closely to functional groups with similar foraging behaviour and floral preferences (Cirtwill et al. 2023).

We counted flowers in the experimental plots at the peak flowering times in 2020 (26 July–3 August) and in 2023 (9–13 July). The counts were based on flower units, defined as approximately 1 cm^2^ of flower surface, with at least one open flower (Carvalheiro et al. 2014). We first counted flower units within 10 randomly placed 0.5 × 0.5 m quadrats; infrequent species that did not occur within the ten small quadrats were counted from the entire 5 × 5 m plot area. We later combined the flower counts and presented them on a square meter scale. The mean total flower abundance per plot was averaged across the years (for further details, see Johanson et al. 2025).

All statistical analyses were conducted in R software (version 4.4.2; R Core Team 2024). To avoid overly complex models, we performed analyses separately for the two sites unless otherwise stated. Treatments were categorized into four categories: Control, Fence, NPK and NPK + Fence. We calculated insect family richness, family‐level Shannon diversity (based on family‐level interactions using the *diversity* function, *vegan*; Oksanen et al. 2025), the total number of recorded interaction events, and network metrics for each plot. The total number of interactions corresponds to the total number of flower‐visitor individuals. We further constructed flower‐visitor interaction matrices for each block × treatment combination using the *framw2webs* function (*bipartite*; Dormann et al. 2008). For each network, we extracted selected network‐level metrics (weighted connectance, specialization (H_2_′) and nestedness) using the *networklevel* function. Connectance measures the proportion of realised interactions among all the possible ones in a network and gives the probability that any pair of species interacts in the network whereas weighted connectance includes also strength or magnitude of the interactions (van Altena et al. 2016). Nestedness describes the tendency for specialists (species with few interaction partners) to interact with a subset of partners used by generalists, such that rare species interact with common species, and common species with both common and rare species (Bascompte et al. 2003; Bascompte and Jordano 2007; Blüthgen et al. 2008). Specialization (H_2_) quantifies how specialized interactions are at the network level (Blüthgen et al. 2008). We summarized metrics by treatment and site and calculated standard errors. Blüthgen's d'specialization index was calculated from the plant‐pollinator interaction matrices to quantify separately plant species‐level and insect family‐level specialization at plot level. We used d′ values to evaluate relationships between species richness or Shannon diversity and specialization across the two grassland types and to test the effects of experimental treatments on plant and insect specialization. Frequency tables and bar plots were used to visualize the relative abundance of insect families and plant species across treatments.

We tested treatment effects on response variables using linear mixed‐effects models (*lme4*; Bates et al. 2015) with grazing exclusion (Fence), nutrient addition (NPK) and their interaction as fixed effects, and block as a random effect. Insect family richness was log‐transformed to improve normality. We checked model assumptions using simulation‐based residual diagnostics (DHARMa). Model simplification was performed via log likelihood ratio tests (Crawley 2012). Model summaries were generated with the *tab_model* function (*sjPlot*; Lüdecke 2025) and pairwise comparisons were conducted using Tukey‐adjusted contrasts (*emmeans*; Lenth and Piaskowski 2025).

To illustrate treatment effects on flower‐visitor community composition, we performed non‐metric multidimensional scaling (NMDS) using *metaMDS* (*vegan*) with Jaccard dissimilarities and up to 20 random starts. Ordination plots were visualized with treatment‐ or site‐level hulls. Differences in community composition among treatments were assessed using permutational multivariate analysis of variance (PERMANOVA; *adonis2*; *vegan*; Oksanen et al. 2025) with 999 permutations.

## 3 Results

### 3.1 General Findings

Across all plots sampled in 2022, we recorded 2569 individuals of flower‐visiting insects (2275 in the montane grassland and 294 in the tundra grassland). Most visitors belonged to Diptera (2444 individuals), followed by Hymenoptera (87), Coleoptera (26), Lepidoptera (8), Hemiptera (2), and Thysanoptera (1). In total, flower‐visitors represented 45 insect families interacting with 20 flowering plant taxa.

### 3.2 Family Richness and Diversity

**FIGURE 1:**
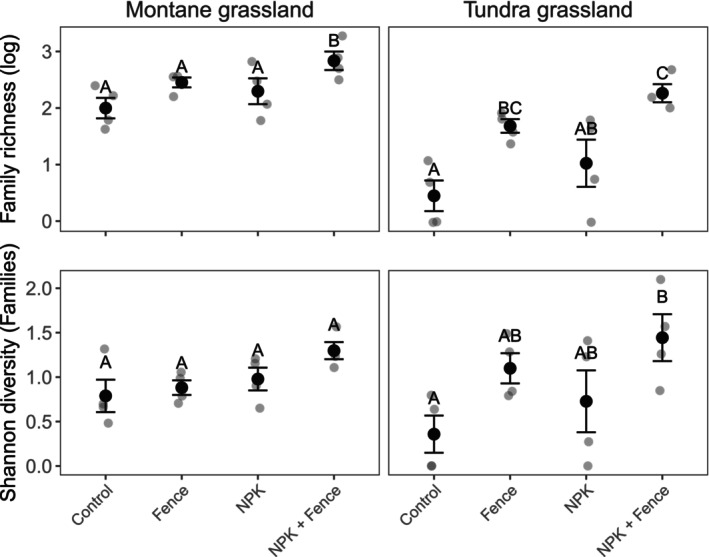
Effects of nutrient addition (NPK) and grazing exclusion (Fence) on flower‐visitor family richness (log‐transformed) and Shannon diversity, in the montane grassland and tundra grassland. Black dots represent treatment means (±SE) and grey circles individual plots. Different letters indicate significant differences among treatments within each site based on post hoc comparisons (emmeans contrasts *p* < 0.05; Supporting Information). For statistical details see Tables 1 and 2.

In the montane grassland, grazing exclusion increased flower‐visitor family richness, while nutrient addition showed a positive but non‐significant effect (Figure 1 and Table 1). Family richness was highest when grazing exclusion and nutrient addition were combined, and this treatment differed significantly from the unfenced, unfertilized control plots (emmeans contrasts: *p* = 0.027; Supporting Information). In the tundra grassland, both grazing exclusion and nutrient addition increased family richness (Figure 1 and Table 1). The strongest increase occurred under the joint grazing exclusion and nutrient addition treatment (emmeans contrasts: *p* = 0.002, Supporting Information), with fenced plots consistently supporting higher richness than grazed plots regardless of nutrient addition.

**TABLE 1:** Simplified mixed model summary of log transformed family richness (non‐significant interaction terms removed).

	Montane grassland			Tundra grassland		
	Estimates	CI	*p*	Estimates	CI	*p*
*Predictors*						
(Intercept)	1.98	1.66 to 2.30	**< 0.001**	0.45	−0.06 to 0.95	0.076
Fence	0.50	0.13 to 0.86	**0.012**	1.24	0.70 to 1.77	**< 0.001**
NPK	0.34	−0.03 to 0.71	0.065	0.58	0.04 to 1.11	**0.037**
*Random effects*						
*σ* ^2^	0.11			0.24		
*τ*_00_	0.00_Blocks_			0.03_Blocks_		
ICC				0.12		
*N*	4_Blocks_			4_Blocks_		
Observations	16			16		
Marginal *R* ^2^/Conditional *R* ^2^	0.466/NA			0.649/0.690		

*Note:* Significant effects (*p* < 0.05) are shown in bold.

In the montane grassland, nutrient addition increased Shannon diversity of flower‐visitor families (Figure 1 and Table 2), whereas grazing exclusion alone had no significant effect. Diversity tended to be highest when grazing exclusion and nutrient addition were combined, although this treatment did not differ significantly from the controls (emmeans contrasts: *p* = 0.072; Supporting Information).

**TABLE 2:** Simplified mixed model summary of Shannon diversity (non‐significant interaction terms removed).

	Montane grassland			Tundra grassland		
	Estimates	CI	*p*	Estimates	CI	*p*
*Predictors*						
(Intercept)	0.73	0.49 to 0.97	**< 0.001**	0.36	−0.11 to 0.83	0.116
Fence	0.21	−0.07 to 0.49	0.133	0.73	0.19 to 1.27	**0.013**
NPK	0.30	0.02 to 0.58	**0.036**	0.36	−0.19 to 0.90	0.175
*Random effects*						
*σ* ^2^	0.06			0.24		
*τ*_00_	0.00_Blocks_			0.00_Blocks_		
*N*	4_Blocks_			4_Blocks_		
Observations	16			16		
Marginal *R* ^2^/Conditional *R* ^2^	0.356/NA			0.419/NA		

*Note:* Significant effects (*p* < 0.05) are shown in bold.

In the tundra grassland, grazing exclusion increased Shannon diversity, with the highest values under the combined treatment (emmeans contrasts: *p* = 0.046; Supporting Information).

### 3.3 Flower‐Visitor Community Composition: Differences Between Sites and Treatment Effects

The non‐metric multidimensional scaling (NMDS) ordination revealed clear differences in flower‐visitor community composition between the sites (Figure S1). This pattern was confirmed by PERMANOVA (*F* = 5.30, *R* ^2^ = 0.15, *p* = 0.001), indicating that site differences explained approximately 15% of the variation in family‐level community structure. The tundra grassland exhibited greater dispersion among plots whereas insect communities in the montane grassland were more tightly clustered, indicating lower among‐plot compositional variability (Figure S1). The montane grassland supported higher family diversity than the tundra grassland, and was dominated by Diptera, particularly Muscidae (75%), with additional contributions from Empididae, Culicidae, Lauxaniidae, and Simuliidae. Several families were unique to the site, including Lauxaniidae, Bibionidae, Drosophilidae, and Vespidae (Table S1). The tundra grassland supported fewer insect families overall (23), and the community composition was strongly dominated by Muscidae (63% of recorded interactions). Other common families included Phoridae, Staphylinidae, and Dolichopodidae, several of which were rare or absent in the montane grassland. Some families were unique to the tundra grassland, including Noctuidae, Oestridae, and Pterophoridae (Table S2).

**FIGURE 2:**
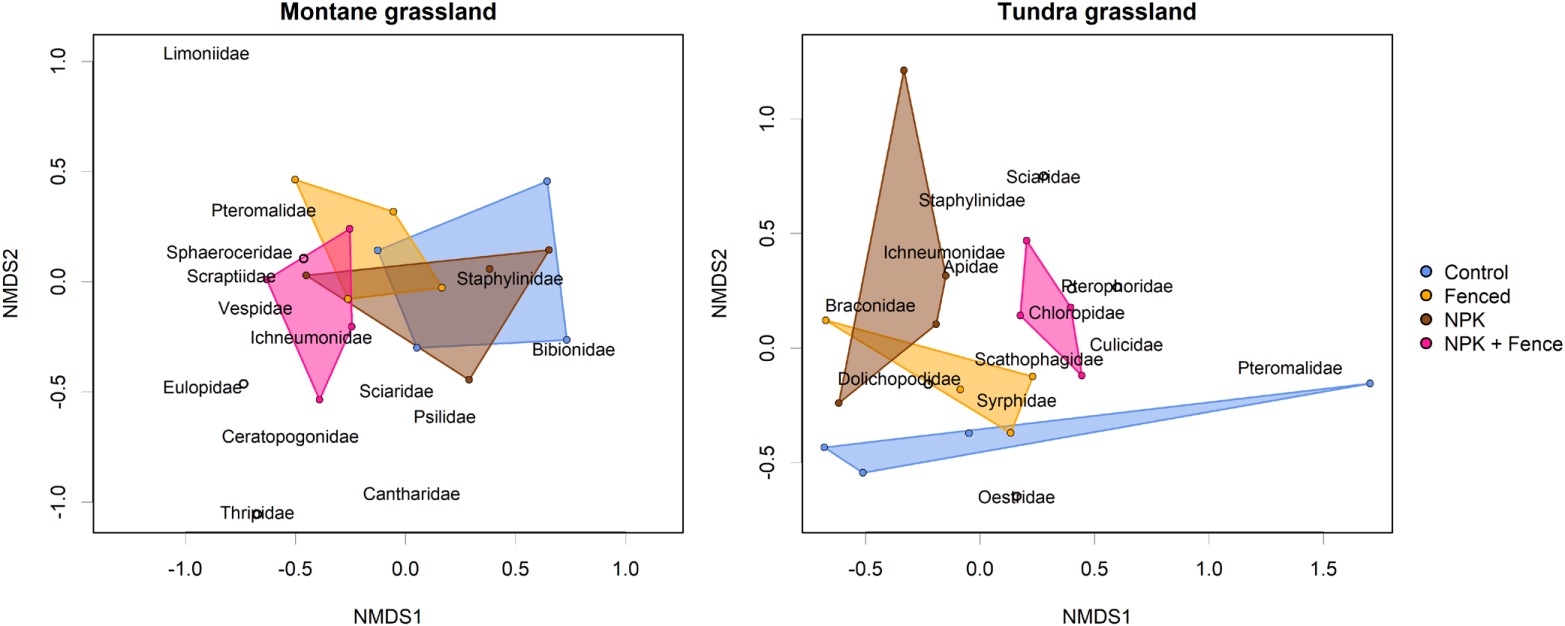
Ordination of flower‐visiting insect community composition (family level) across treatments in the montane grassland and tundra grassland. The NMDS multivariate analysis shows clustering of Control (blue), Fence (orange), NPK (brown), and NPK + Fence (pink) treatments, based on a NMDS multivariate analysis of insect‐visitor abundance data. Polygons enclose treatment groups, with centroids connected by lines. Family names indicate taxa contributing most to differences in community composition among treatments.

In the montane grassland, flower‐visitor community composition was significantly altered by the combined effects of grazing exclusion and nutrient addition (PERMANOVA *F* = 3.0, *R* ^2^ = 0.17, *p* = 0.006), whereas single‐factor treatments had weaker or non‐significant effects. Grazing exclusion and nutrient addition explained 17% of the variation in community composition. Control and NPK plots clustered closely in ordination space, indicating broadly similar community composition, while fenced plots, especially fenced NPK plots, were more distinct (Figure 2). Treatment‐specific associations were evident in the montane grassland (Table S1). Control plots were associated with diverse taxa such as Culicidae, Simuliidae, and Sciaridae. Families such as Culicidae, Bibionidae, Dolichopodidae, and Scatophagidae were associated with nutrient addition, whereas Apidae, Empididae, Braconidae, Lauxaniidae, and Simuliidae were more frequent in fenced plots (Table S1). Fenced NPK plots were characterized by a broader suite of families, including Bibionidae, Empididae, Lauxaniidae, and Phoridae, as well as several rarer taxa such as Cecidomyiidae, Ceratopogonidae, Limoniidae, and Thripidae, indicating a shift towards a more compositionally distinct and taxonomically diverse visitor assemblage (Figure 2 and Table S1).

In the tundra grassland, community composition differed significantly among treatments and the strongest compositional shift occurred under fenced NPK treatment, while single‐factor treatments showed weaker effects (PERMANOVA, *F* = 2.6, *R* ^2^ = 0.15, *p* = 0.012; Figure 2). Nutrient addition and grazing exclusion together explained 15% of the variation in family‐level community structure. Control plots were characterized primarily by Muscidae (which were proportionally in highest numbers here), Chironomidae, Chloropidae and Syrphidae, which occurred mainly or only in the controls. NPK plots were associated with Dolichopodidae, Sciaridae and Staphylinidae. Fenced plots without NPK addition were mainly associated with Dolichopodidae, Phoridae and Simuliidae, and the combined NPK + Fence treatment showed the strongest compositional shift and was characterized by Ichneumonidae, Phoridae and Staphylinidae, as well as several rare families like Noctuidae, Tephritidae and Zygenidae.

Weighted interaction networks illustrate how treatment‐driven changes in community composition translate into differences in interaction structure at both sites (Figures S2 and S3). Across all treatments, in the montane grassland, interactions were concentrated on a few flowering plant species. *Geranium sylvaticum* consistently received the highest number of visits, followed by *Anthriscus sylvestris* and *Ranunculus subborealis* (Table S3). Networks in the grazed control plots were dominated by Muscidae, Culicidae, and Empididae visiting mainly *Geranium sylvaticum* and *Ranunculus subborealis* (Figure S2a), suggesting open vegetation with widely available generalist flowers. These networks comprised a small number of strong links and low taxonomic breadth, reflecting the strong dominance of Muscidae (Figure S2). Grazing exclusion strengthened the links between *Anthriscus sylvestris* and *Geranium sylvaticum* and Empididae, while Apidae emerged as additional visitors and Muscidae remained abundant (Figure S2a). Under unfenced NPK enrichment, *Ranunculus subborealis* remained the dominant floral species, but Empididae, Culicidae, and Simuliidae became more common visitors alongside Muscidae (Figure S2b), likely reflecting changes in flower resources and microhabitat structure. In fenced NPK plots, interactions were dominated by *Anthriscus sylvestris* , *Geranium sylvaticum* , and *Trollius europaeus* and involved a broader suite of visiting families including Empididae, Phoridae, and Lauxaniidae, with occasional Syrphidae and Vespidae (Figure S2b and Table S3).

In the tundra grassland, interaction networks also revealed a strong treatment‐dependent restructuring (Figure S3). Across all treatments, interactions were concentrated on a few flowering plant species, notably *Hieracium* sect. *Alpina* and *Solidago virgaurea*, which together accounted for most visits. Other species such as *Bistorta vivipara* , *Ranunculus subborealis*, and *Rhodiola rosea* contributed relatively few interactions (Table S4). Control (grazed) networks were extremely simple and dominated almost exclusively by Muscidae visiting *Hieracium* sect. *Alpina* and *Solidago virgaurea* (Figure S3). These networks contained very few strong links, consistent with the strong Muscidae dominance observed in both NMDS ordination and frequency table (Figure 2 and Table S2). Grazing exclusion alone increased the number of interactions and modestly broadened network structure (Figure S3). Under unfenced NPK treatment, networks remained relatively plant‐poor but showed a clearer diversification of visiting families. Fenced plots showed stronger links between *Hieracium* sect. *Alpina* and *Solidago virgaurea* and a wider suite of Dipteran families, including Dolichopodidae, Phoridae, and Simuliidae, although Muscidae remained the dominant visitors (Figure S3). While *Hieracium* sect. *Alpina* continued to receive a large proportion of visits, interactions increasingly involved Dolichopodidae, Sciaridae, and Staphylinidae, in addition to Muscidae, corresponding to their higher relative abundance in the frequency table (Table S2). The strongest restructuring occurred under fenced NPK treatment, where interaction networks expanded markedly in size and taxonomic breadth (Figure S3). These plots were characterized by very strong interaction links involving *Hieracium* sect. *Alpina* and *Solidago virgaurea* and, but also by the involvement of a wide array of visitor families, including Chironomidae, Ichneumonidae, Phoridae, and Staphylinidae, together with several rare taxa such as Noctuidae, Tephrididae, and Zygaenidae.

### 3.4 Number of Interactions in Plant‐Pollinator Networks

**FIGURE 3:**
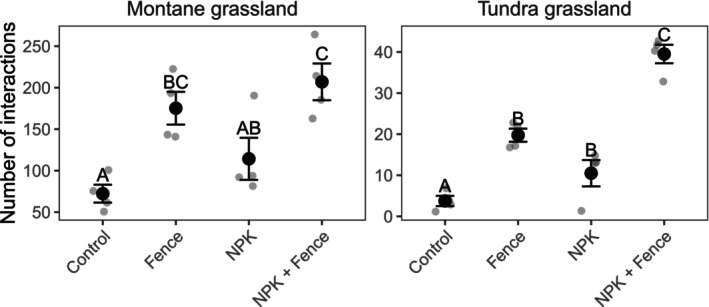
Effects of grazing exclusion (Fence), nutrient addition (NPK), and their combination (NPK + Fence) on the total number of interactions in the montane grassland and in the tundra grassland. Black dots represent treatment means (±SE) and grey circles individual plots. Different letters indicate significant differences among treatments within each site based on post hoc comparisons (emmeans contrasts *p* < 0.05; Supporting Information).

Across all plots, the number of plant‐pollinator interactions was substantially higher in the montane grassland (2275) than in the tundra grassland (294) (Tables S1 and S2). Because interactions represent observed flower visitation events, they reflect abundance and activity of flower‐visiting insects within the experimental plots. In the montane grassland, grazing exclusion strongly increased the number of interactions, rising from 289 interactions in control plots to 708 in fenced plots. Nutrient addition showed a positive but non‐significant effect (457 interactions), while the combined treatments produced the highest number of interactions (828), indicating additive effects between the treatments (Figure 3 and Table 3, emmeans contrast: *p* = 0.001; Supporting Information). In the tundra grassland, grazing exclusion and nutrient addition acted synergistically to produce the highest number of interactions (158), followed by grazing exclusion (79 interactions), NPK treatment (42 interactions), and control plots (15 interactions) (Figure 3 and Table 3; Supporting Information). Tukey post hoc contrasts confirmed that all treatment combinations differed significantly from the control plots (all emmeans contrasts: *p* < 0.05; Supporting Information).

**TABLE 3:** Number of interactions of simplified (montane grassland) and full (tundra grassland) mixed model summary.

	Montane grassland			Tundra grassland		
	Estimates	CI	*p*	Estimates	CI	*p*
*Predictors*						
(Intercept)	74.81	36.06 to 113.56	**0.001**	3.75	−1.16 to 8.66	0.120
NPK	36.87	−1.75 to 75.50	0.059	6.75	2.32 to 11.18	**0.007**
Fence	97.88	59.25 to 136.50	**< 0.001**	16.00	11.57 to 20.43	**< 0.001**
NPK × Fence				13.00	6.73 to 19.27	**0.001**
*Random effects*						
*σ* ^2^	1231.66			7.92		
*τ*_00_	316.14_Blocks_			11.54_Blocks_		
ICC	0.20			0.59		
*N*	4_Blocks_			4_Blocks_		
Observations	16			16		
Marginal *R* ^2^/Conditional *R* ^2^	0.653/0.724			0.908/0.963		

*Note:* Significant effects (*p* < 0.05) are shown in bold.

### 3.5 Network Structure (Connectance, Specialization and Nestedness)

**FIGURE 4:**
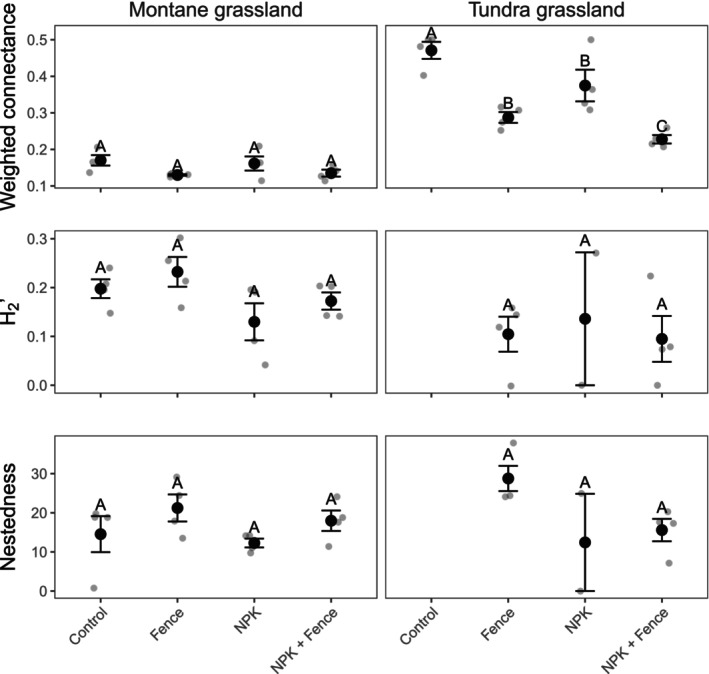
Effects of grazing exclusion (Fence), nutrient addition (NPK), and their combination (NPK + Fence) on weighted connectance, standardized plant‐pollinator network‐level specialization index H_2_′, and nestedness, in the montane and tundra grassland. Black dots represent treatment means (±SE) and grey circles individual plots. Different letters indicate significant differences among treatments within each site based on post hoc comparisons (emmeans contrasts *p* < 0.05; Supporting Information).

In the montane grassland, network structure responded modestly to the experimental treatments (Figure S2, Tables 4 and 5). Due to low flower species richness in some control plots in the tundra grassland, unweighted connectance was constrained by network size and showed little variation among treatments. To better capture changes in interaction structure, we focused on weighted connectance, which is less sensitive to differences in network size. In the montane grassland, grazing exclusion reduced weighted connectance of the flower‐visitor network (Figure 4 and Table 4). Pairwise contrasts indicated lower weighted connectance in fenced plots than in grazed controls (emmeans contrasts: *p* = 0.09; Supporting Information) and in fenced NPK plots relative to unfenced NPK plots (emmeans contrasts: *p* = 0.09; Supporting Information), although these differences were not statistically significant. At the same time, nutrient addition reduced network specialization (H_2_′), indicating a shift towards more generalized interactions (Figure 4 and Table 5). Nestedness showed no significant response to grazing exclusion, although mean values tended to be higher in fenced plots (Figure 4 and Table 5).

**TABLE 4:** Simplified mixed model summary of weighted connectance (non‐significant interaction terms removed).

	Montane grassland			Tundra grassland		
	Estimates	CI	*p*	Estimates	CI	*p*
*Predictors*						
(Intercept)	0.17	0.14 to 0.19	**< 0.001**	0.46	0.41 to 0.51	**< 0.001**
NPK	−0.00	−0.03 to 0.03	0.885	−0.08	−0.13 to −0.02	**0.011**
Fence	−0.03	−0.06 to −0.01	**0.022**	−0.17	−0.22 to −0.11	**< 0.001**
*Random effects*						
*σ* ^2^	0.00			0.00		
*τ*_00_	0.00_Blocks_			0.00_Blocks_		
ICC	0.02			0.04		
N	4_Blocks_			4_Blocks_		
Observations	16			16		
Marginal *R* ^2^/Conditional *R* ^2^	0.317/0.328			0.769/0.778		

*Note:* Significant effects (*p* < 0.05) are shown in bold.

**TABLE 5:** Simplified mixed effects model summary of H_2_′ and nestedness in the montane grassland (non‐significant interaction terms removed).

Predictors	H2′			Nestedness		
	Estimates	CI	*p*	Estimates	CI	*p*
(Intercept)	0.20	0.14 to 0.25	**< 0.001**	14.71	8.89 to 20.52	**< 0.001**
NPK	−0.06	−0.12 to −0.00	**0.039**	−2.58	−9.30 to 4.13	0.415
Fence	0.04	−0.02 to 0.10	0.188	6.17	−0.55 to 12.88	0.068
NPK × Fence						
*σ* ^2^	0.00	37.91				
*τ*_00_	0.00_Blocks_	0.00_Blocks_				
ICC						
*N*	4_Blocks_			4_Blocks_		
Observations	16			16		
Marginal *R* ^2^/Conditional *R* ^2^	0.332/NA			0.247/NA		

*Note:* Significant effects (*p* < 0.05) are shown in bold.

In the tundra grassland, both grazing exclusion and nutrient addition reduced weighted connectance (Figure 4 and Table 4). The strongest reduction occurred under the combined treatment, which differed clearly from all other treatments (emmeans contrasts: *p* < 0.0002; Supporting Information). Treatment effects explained a large proportion of the variation in weighted connectance in the tundra grassland (marginal *R* ^2^ = 0.769, conditional *R* ^2^ = 0.778), whereas explained variance was considerably lower in the montane grassland (marginal *R* ^2^ = 0.317, conditional *R* ^2^ = 0.328). In the tundra grassland, interpretation of network metrics was limited by the extremely simple structure of control networks. Specialization (H_2_′) and nestedness could not be quantified reliably, although overall specialization appeared lower than in the montane grassland (Figure 4).

Species‐ or family‐level specialization (Blüthgen's d′) showed high variability across treatments in both grassland types (Figure S4). In the montane grassland, plant's d´ values were consistently low and did not differ among treatments (Figure S4 and Table 6), whereas insect d´ decreased significantly under nutrient addition (*p* = 0.002) and grazing exclusion (*p* = 0.03, Table 6). Pairwise comparisons confirmed lower insect specialization in NPK plots (*p* = 0.013) and in the combined NPK + Fence treatment (*p* = 0.002) compared to controls.

**TABLE 6:** Simplified mixed effects model summary of Blüthgen's d′ values in the montane grassland (non‐significant interaction terms removed).

Montane grassland	Plants			Insects		
	Estimates	CI	*p*	Estimates	CI	*p*
*Predictors*						
(Intercept)	0.17	0.10 to 0.25	**< 0.001**	0.22	0.17 to 0.27	**< 0.001**
NPK	−0.07	−0.15 to 0.01	0.104	−0.08	−0.13 to −0.03	**0.002**
Fence	−0.01	−0.09 to 0.08	0.900	−0.06	−0.11 to −0.01	**0.030**
*Random effects*						
*σ* ^2^	0.04			0.03		
*τ*_00_	0.00_Block_			0.00_Block_		
*N*	4_Block_			4_Block_		
Observations	96			192		
Marginal *R* ^2^/Conditional *R* ^2^	0.028/NA			0.072/NA		

*Note:* Significant effects (*p* < 0.05) are shown in bold.

In the montane grassland, d′ values increased significantly with flower species richness (*R* ^2^ = 0.54, *p* = 0.001; Figure S5) and showed a non‐significant positive relationship with Shannon diversity (*R* ^2^ = 0.17, *p* = 0.115; Figure S6). In the tundra grassland, treatment effects on d′ were not formally tested because d′ was fixed to zero in control plots. However, d′ showed a significant negative relationship with Shannon diversity (*R* ^2^ = 0.50, *p* = 0.007) and no relationship with flower species richness (*R* ^2^ = 0.01, *p* = 0.7; Figure S6).

### 3.6 Relationship Between Flower Resource Availability With Flower‐Visitor Assemblages

**FIGURE 5:**
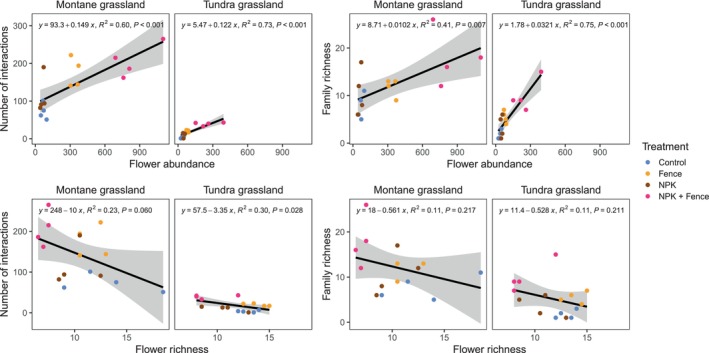
The relationships between flower resources and flower‐visitor responses in the montane and tundra grasslands. Upper panels show the relationship between flower abundance and number of interactions (left) and insect family richness (right). Lower panels show the relationship between flower richness and number of interactions (left) and insect family richness (right). Points represent individual plots coloured by treatment (Control, Fence, NPK, NPK + Fence) and solid lines indicate linear regression with 95% confidence intervals (shaded areas). Regression equations, coefficients of determination (*R* ^2^), and significance levels are shown within each panel.

Across all plots, flower‐visitor abundance increased with flower abundance in both the montane and the tundra grassland, with the relationship being stronger in the tundra (Figure 5, top panels). Similarly, flower‐visitor family richness increased with flower abundance, although the response was weaker in the montane grassland. In contrast, flower‐visitor abundance declined with increasing flower richness, especially in the tundra grassland, whereas flower‐visitor richness showed no significant relationship with flower richness in either system (Figure 5, bottom panels).

## 4 Discussion

Our findings demonstrate that grazing and nutrients are important regulators of flower‐visiting insect communities and plant‐pollinator interaction networks in Arctic grasslands. Responses were consistently stronger in the low‐productivity tundra grassland than in the high‐productivity montane grassland. Across both systems, grazing exclusion emerged as the primary driver of flower‐visitor richness, number of interactions, and network structure, while nutrient addition played a secondary, context‐dependent role. These findings highlight how indirect top‐down effects through grazing‐mediated changes in vegetation and bottom‐up nutrient enrichment can regulate pollination processes in Arctic and sub‐Arctic ecosystems, with outcomes dependent on ecosystem productivity.

### 4.1 Effects on Flower‐Visitor Richness and Diversity

As predicted, grazing exclusion increased flower‐visitor family richness in both grasslands, with particularly strong effects in the tundra grassland, supporting our hypothesis that increased floral resource availability promotes richer flower‐visitor communities. This indicates that current grazing pressure constrains pollinator communities across sites, and these effects are especially strong in nutrient‐poor tundra grassland where floral resources are inherently scarce (Johanson et al. 2025; Vasilevskaya 2025). Nutrient addition alone had limited effects on visitor richness, consistent with findings of Wang et al. (2022), whereas its combination with grazing exclusion enhanced richness. This pattern supports the idea that nutrient resource limitation constrains flower‐visitor richness more strongly in nutrient‐poor systems, whereas in more productive grasslands grazing exclusion seems to be the primary constraint and additional nutrient inputs offer limited benefits unless grazing pressure is simultaneously reduced. As shown by Johanson et al. (2025) grazing exclusion and nutrient addition enhanced floral abundance, and nutrients further increased floral resource availability and quality, together supporting a broader range of pollinator taxa.

In partial agreement with our hypothesis that nutrient addition would increase flower‐visitor diversity where it enhances floral resources, Shannon diversity responded to nutrient addition in the montane grassland, suggesting that even in more productive systems, changes in floral resource quality or quantity can influence flower‐visitor community structure, although overall responses remained modest and largely stable across treatments. This may indicate that pollinator communities in productive systems with high pollinator density and abundant floral resources already operate near saturation point, where additional resource input or grazer removal offers limited benefits for them. Such patterns align with evidence that pollinator richness saturates with increasing floral resources and pollination dynamics become more stable at higher levels of resource availability (Ebeling et al. 2008). The stronger diversity responses in tundra communities suggest that pollinator assemblages in resource‐limited systems remain far below resource saturation and therefore respond more strongly to increases in floral resources than communities in productive grasslands. This suggests that while nutrient enrichment alone has limited impact on visitor assemblages, it can additively enhance diversity when grazing pressure is removed. Together these findings reinforce that in resource‐limited systems, pollinator richness and diversity are more sensitive and responsive to changes in floral resources, whereas in more productive grasslands, diversity responses are subtler and more buffered by existing resource availability.

### 4.2 Effects on Flower‐Visitor Assemblage Composition

The two grasslands differed in their baseline community composition, reflecting contrasting environmental conditions, productivity, and floral resource availability. The montane grassland supported more taxonomically diverse but compositionally stable insect communities, whereas tundra grassland assemblages were less diverse but more variable and responsive to treatments. Consistent with our prediction that grazing exclusion and nutrient addition reshape flower‐visitor assemblages through changes in floral community structure, flower‐visitor assemblage composition responded most strongly to the combined grazing exclusion and nutrient addition (NPK + Fence) at both sites, whereas single‐factor treatments had weaker effects. Across treatments, communities were dominated by Diptera, reflecting their well‐documented key role as pollinators in Arctic and alpine systems (Olesen et al. 2008; Zoller et al. 2023). Nutrient addition increased the representation of families associated with nutrient‐rich or decomposer pathways such as Empididae and Drosophilidae, paralleling findings by Veen et al. (2024), that fertilization increased detritivores in high‐biomass systems. The higher representation of bees and parasitoid wasps such as Apidae and Braconidae, as well as other pollinating taxa such as Empididae, Muscidae, Lauxaniidae, and Syrphidae in fenced plots, suggests that increased vegetation height and floral density created more favorable foraging and nesting conditions for taxa that were limited in heavily grazed systems. The fenced NPK treatment (NPK + Fence) supported the highest taxonomic diversity, dominated by different Dipteran families, parasitoid wasps, hoverflies, as well as a diverse assemblage of other taxa. This reflects that enhanced floral resource quality and availability (Johanson et al. 2025) and habitat complexity together promoted more heterogeneous visitor assemblages, allowing taxa with different ecological requirements to coexist.

In the tundra grassland, control plots were dominated by Chironomidae, Muscidae, and Pteromalidae, taxa adapted to open, grazed habitats characterized by sparse wind‐exposed flowers and detrital substrates (Willemstein 1987). Fenced plots formed a distinct cluster that, apart from Muscidae, was associated with Phoridae and Syrphidae, groups that may benefit from taller, more structurally complex flower‐rich vegetation following grazing removal. The NMDS ordination further revealed a clear separation between control and NPK plots, with the latter dominated by parasitoid and potentially pollinating Dipteran families such as Chironomidae, Dolichopodidae, and Staphylinidae. These findings align with Veen et al. (2024) who reported that nutrient addition can reduce the abundance of typical pollinators (bees, syrphids, and lepidopterans) while increasing other taxa. The fenced NPK treatment supported the most diverse assemblage, characterized by families such as Chloropidae, Muscidae, Phoridae, and Staphylinidae. This aligns with the observation that nutrient addition combined with grazing exclusion creates a dense cover of a few highly rewarding key species that concentrates diverse insect visitation (Johanson et al. 2025). Among flowering plants, *Hieracium* sect. *Alpina* consistently functioned as an important interaction hub across treatments, while *Solidago virgaurea* (the most connected plant), *Bistorta vivipara* , and *Ranunculus acris* contributed more strongly under grazing exclusion or nutrient addition, highlighting how a small number of abundant core species can structure Arctic pollination networks (Khorsand et al. 2025).

In control plots, interactions occurred primarily between Muscidae and *Hieracium*, indicating a simplified, functionally constrained system dominated by opportunistic flies. Overall, these results indicate that flower‐visiting insect assemblages in Arctic grasslands are highly sensitive to joint changes in grazing and nutrient availability.

### 4.3 Effects on Number of Interactions and Network Structure

Consistent with our hypothesis that treatments enhancing floral resource would increase network size, grazing exclusion strongly increased the number of plant‐pollinator interactions and thus the abundance of flower‐visitors, as well as network size in both grassland types, highlighting its central role in regulating pollination dynamics. Although treatment effects differed between grasslands, grazing exclusion consistently increased interaction numbers, whereas nutrient addition mainly strengthened responses in the tundra, producing a strong synergistic effect. These patterns mirrored treatment effects on floral resources (Johanson et al. 2025), in which grazing exclusion and nutrient addition strongly increased flower abundance but decreased flower species richness. The observed relationship between floral resources and flower‐visitors indicates that flower‐visitors primarily track overall density of floral resources rather than floral diversity per se. Strong positive relationships between flower abundance and both the number of interactions and family richness were evident, particularly in the tundra grassland. In contrast, flower richness showed weak or negative relationships with flower‐visitor abundance. In Arctic grasslands dominated by opportunistic Dipteran visitors, capable of exploiting a wide range of plant species (Olesen et al. 2008; Zoller et al. 2023), flower density and visibility seem to outweigh floral diversity as drivers of pollinator abundance and diversity. This pattern is consistent with findings that pollinator visitation rates often increase with floral display size and density (Grindeland et al. 2005; Makino et al. 2007). However, contrary to what has been shown in some studies, where higher floral diversity promotes greater pollinator diversity (Fründ et al. 2010), floral diversity did not consistently correspond to higher pollinator diversity in our study.

Despite large increases in number of interactions, network structure responded in more nuanced ways. In line with our prediction that increased floral resources would expand network size while reducing connectance, weighted connectance declined strongly with grazing exclusion in both grasslands and declined further with nutrient addition in the tundra grassland. In the montane grassland, weighted connectance was largely unaffected by nutrient addition, indicating greater structural stability in the high‐productivity system (van Altena et al. 2016). In nutrient‐poor systems, however, effects of enhanced nutrient availability for plants became apparent once grazing pressure was removed and floral resources could accumulate supporting greater pollinator visitation. In the nutrient‐amended grazed plots, floral resources remain scarce, and the few available are efficiently used, increasing weighted connectance. Our results suggest that the low productivity tundra networks are relatively simple and tightly coupled, such that changes in vegetation structure following grazing exclusion cascade rapidly into the flower‐visitor networks. Overall increased visitation does not necessarily lead to more interconnected networks, as mutualistic networks typically are structured by a small number of core generalists and many weak, unevenly distributed interactions (Bascompte and Jordano 2007). Instead, high flower abundance (e.g., in the fenced NPK plots; Johanson et al. 2025) appears to concentrate interactions onto patches of highly rewarding plant species (such as *Anthriscus sylvestris, Geranium sylvaticum, Hieracium* sect. *Alpina* and *Solidago virgaurea*), reducing the proportion of realized links, while low‐abundance patches, even if diverse, support only sparse and stochastic visitation. This effect was strongest in the tundra grassland, where combined treatments caused sharp declines in weighted connectance. As floral resources became abundant but less evenly distributed, pollinators can afford to specialize and become increasingly selective and focused on a few dominant plant species, leading to less connected interaction networks. Similar shifts under grazing pressure have been documented by Rakosy et al. (2022), who found that intensive grazing reduced interaction diversity while increasing specialization and niche overlap, reflecting reorganization of interactions around a few dominant plant species.

Contrary to our prediction that greater floral resource availability would promote more specialized interaction networks, network specialization (H_2_′) showed limited responsiveness to experimental manipulation, with no significant treatment effects, although specialization was numerically lower under nutrient addition. This suggests that even though weighted connectance declined and interactions became concentrated on dominant plant species, flower‐visitors largely maintained generalized foraging patterns and did not strongly reorganize partner selectivity. However, slight differences in specialization between NPK fertilized plots and fenced plots suggest that grazing may indirectly matter for specialization via flowering plant composition and height (Yan et al. 2026). Increased vegetation height and floral diversity may promote niche partitioning among pollinators, whereas nutrient enrichment may homogenize floral resources and reduce opportunities for specialization. Because H_2_’ reflects specialization of both plants and pollinators, dominance by a few abundant plant species may also constrain the range of possible interaction partners and thereby limit detectable variation in specialization. In the tundra grassland, specialization could not be quantified for control plots, due to extremely low network complexity, precluding formal tests of treatment effects. H_2_′ values were generally lower than in the montane grassland, consistent with observed dominance by generalist Dipteran visitors. This pattern reflects the inherently generalist nature of the system (Elberling and Olesen 1999), where pollinator communities rely on a small set of available floral resources (Khorsand et al. 2025).

Species‐and family‐level specialization (d′) also showed generally weak responses to treatments, particularly in the montane grassland. Increased floral resources did not lead to stronger niche partitioning. Instead, insect specialization decreased under both nutrient addition and grazing exclusion with the strongest reduction under the combined treatment. When floral resources become dominated by fewer plant species, despite overall abundance, pollinator interactions may become more generalized due to increased overlap in resource use, which can reduce network‐level specialization (d′), consistent with patterns observed under reduced floral diversity (Gómez‐Martínez et al. 2022).

In contrast, plant‐level specialization remained low and unresponsive to treatments, indicating that plants maintained generalized interaction patterns regardless of changes in grazing or nutrients. This asymmetry suggests that pollinators are more flexible than plants in adjusting interaction patterns in response to environmental change. This generally limited responsiveness to experimental manipulation is consistent with Fründ et al. (2010), who found that increased diversity does not necessarily lead to higher specialization in plant‐pollinator networks. However, diversity‐specialization relationships differed between grasslands. In the montane grassland, d′ increased strongly with flower richness, indicating that diverse floral resources promote greater partner selectivity suggesting that resource diversity is a key driver of specialization. In contrast the lack of relationship with Shannon diversity suggests a weaker role for evenness. In the tundra grassland, specialization declined with increasing Shannon diversity and showed no relationship with species richness, indicating that when diversity increases, interactions remain dominated by generalist foraging strategies. These patterns imply, that in simpler tundra systems, biotic constraints and generalist strategies limit the extent to which specialization can respond to treatments. d′ values were also generally lower in the tundra grassland, particularly among insects, reflecting dominance by highly mobile and opportunistic Diptera, whereas slightly higher plant d´ in the montane system may reflect greater floral resource stability and longer growing season, allowing for slightly more fine‐tuned plant‐visitor associations. However, given limited variation in the tundra dataset, these patterns should be interpreted cautiously.

Contrary to our prediction that greater flower abundance would increase network nestedness, this metric showed little response to treatments, although it was higher under grazing exclusion in the montane site, consistent with increased network complexity and potential robustness to species loss. Data limitations prevented formal testing in the tundra grassland, but descriptive patterns suggest greater sensitivity to both grazing and nutrient enrichment in nutrient‐limited ecosystems.

Taken together, our results show that nutrient addition and grazing exclusion primarily altered the density and distribution of plant‐pollinator interactions rather than the underlying network structure. Grazing exclusion and nutrient addition increased the number of realized interactions (C) and promoted more generalized interaction patterns but produced only modest changes in partner selectivity (H_2_′) and nestedness. Pollinator communities therefore appear to respond to disturbance by adjusting the number of interactions rather than fundamentally reorganizing plant associations, consistent with previous work showing nutrient enrichment can increase visitation rates without substantially altering network‐level metrics (Burkle and Irwin 2009). Such structural stability may help buffer pollination processes even when interaction intensity fluctuates. These results further suggest that pollination network specialization may be more dynamic in resource‐limited systems and more resistant to change in more productive systems.

### 4.4 Context Dependency and Implications for Arctic Grasslands

Our results demonstrate that grazing and nutrient enrichment can substantially alter the structure and potential stability of pollination systems in Arctic grasslands. In the montane grassland, responses emerged mainly under combined treatments that promoted richer and more complex networks, involving changes in the number of interactions rather than fundamental reorganization of network structure, which indicates greater resilience in more productive systems. In contrast, tundra grasslands where networks are naturally small and highly generalized exhibited consistently stronger responses. In these low‐productivity systems, small shifts in vegetation structure and resource availability rapidly cascade through pollinator communities and interaction networks, altering richness, composition, and network structure. These effects can further propagate through the endotherm food webs by regulating availability of insects as food for insectivorous birds; thereby, trophic interactions (reviewed by Filazzola et al. 2020) extend to broader Arctic trophic interactions.

Although the experimental manipulations increased the number of interactions, they reduced weighted connectance, potentially increasing vulnerability to future disturbances if interactions become concentrated on a few dominant species. The extremely depauperate visitor networks observed in the ambient (Control) tundra grassland highlight the scarcity of flower resources at current grazing regimes, raising concerns about the long‐term sustainability of pollination services in Arctic grasslands under ongoing global change. Our results suggest that, if current grazing regimes persist, pollination networks will become increasingly simplified, dominated by relatively few generalist taxa with reduced functional redundancy and greater vulnerability to future disturbances. These effects are likely to be amplified or modified by other global change drivers already affecting Arctic ecosystems, including climate warming, shrub expansion, nutrient enrichment, and altered phenology (Box et al. 2019), which may increase competition for a decreasing number of pollinators (Tiusanen et al. 2020), contribute to nectar depletion (Sponsler et al. 2024), and push already simplified pollination systems closer to ecological tipping points. Future studies should assess how interacting global change drivers influence pollination networks over the long term and whether these changes reduce plant reproductive dynamics, pollinator persistence, and ecosystem resilience. Because many Arctic plants rely on insect pollination (Rodger et al. 2021), sustained declines in pollinator communities could reduce plant reproduction with potential cascading consequences for ecosystem functioning.

These results call for ecosystem‐specific management approaches that consider the structure of species interactions when predicting impacts of multiple global change factors on pollination services (Vanbergen and Insect Pollinators Initiative 2013). Management strategies that preserve abundant floral resources and spatial heterogeneity in grazing intensity are important for supporting insect communities with different habitat requirements. Rotational or late‐season grazing rather than continuous grazing may therefore be critical for conserving pollination processes in Arctic landscapes (Vulliamy et al. 2006; Sjödin 2007).

## Author Contributions

**Harry Olde Venterink:** conceptualization, investigation, writing – review and editing, methodology. **Martin Andrzejak:** data curation, investigation, formal analysis, writing – review and editing, visualization, methodology. **Luísa G. Carvalheiro:** conceptualization, methodology, writing – review and editing, investigation. **Risto Virtanen:** conceptualization, investigation, funding acquisition, writing – review and editing, visualization, methodology, formal analysis, project administration, resources, supervision, data curation. **Nicolina Johanson:** funding acquisition, investigation, conceptualization, writing – original draft, writing – review and editing, visualization, formal analysis, data curation. **Anu Eskelinen:** investigation, funding acquisition, writing – review and editing, supervision, project administration, resources.

## Funding

The research has been funded by the following foundations. Societas pro Fauna et Flora Fennica, Nordenskiöldsamfundet, University of Oulu (ArcI grant), Victoriastiftelsen and Svenska kulturfonden. Academy of Finland (#29719), Waldemar von Frenckells stiftelse. Kilpisjärvi biological station supported the fieldwork.

## Disclosure

*Permits*: The permit for the field experiment was granted by Metsähallitus (nos. MH2812/2019 and MH1916/2019).

## Conflicts of Interest

The authors declare no conflicts of interest.

## Supporting information


**Figure S1:** An ordination diagram of flower‐visiting insect community composition (family level) in the tundra (green) and montane grassland (blue). The diagram shows differences in community structure among based on a NMDS multivariate analysis of insect‐visitor abundance data. Polygons enclose treatment groups, with centroids connected by lines. Labels indicate the dominant insect families.
**Table S1:** Frequency table showing the abundance of insect families collected at the montane grassland site across treatments: Control, Fence, NPK, and NPK + Fence. Each value represents the total number of individuals recorded for a given family within each treatment. Total sample sizes for each treatment are shown at the bottom of the plot, and overall family totals are shown to the right.
**Table S2:** Frequency table showing the abundance of insect families collected at the tundra grassland site across treatments: Control, Fence, NPK, and NPK + Fence. Each value represents the total number of individuals recorded for a given family within each treatment. Total sample sizes for each treatment are shown at the bottom of the plot, and overall family totals are shown to the right.
**Table S3:** Frequency table showing the abundance of plant species at the montane grassland site across treatments: Control, Fence, NPK, and NPK + Fence. Each value represents the total number of individuals recorded for a given species within each treatment. Total sample sizes for each treatment are shown at the bottom of the plot, and overall totals for each taxon are shown to the right.
**Table S4:** Frequency table showing the abundance of plant species at the tundra grassland site across treatments: Control, Fence, NPK, and NPK + Fence. Each value represents the total number of individuals recorded for a given species within each treatment. Total sample sizes for each treatment are shown at the bottom of the plot, and overall totals for each taxon are shown to the right.
**Figure S2:** (a) Weighted flower‐visitor networks in the montane grassland constructed for experimental treatments: Control (grazed) and Fence (grazing exclusion). The rectangles on the right side represent plant species and flower‐visitor families on the left side.
**Figure S2:** (b) Weighted flower‐visitor networks in the montane grassland constructed for experimental treatments: NPK (nutrient addition) and NPK + Fence (nutrient addition and grazing exclusion combined). The rectangles on the right side represent plant species and flower‐visitor families on the left side.
**Figure S3:** Weighted flower‐visitor networks in the tundra grassland constructed for each experimental treatment: Control (grazed), Fence (grazing exclusion), NPK (nutrient addition) and Fenced NPK (combined nutrient addition and grazing exclusion). The rectangles on the right side represent plant species and flower‐visitor families on the left side.
**Figure S4:** Relationship between Blüthgen's d′ specialization index for insects (green) and plants (blue) in (a) the montane grassland and (b) the tundra grassland. Points represent mean values for each species (plants) or families (insects) under four experimental treatments: Control, Fence, NPK and NPK + Fence. Large, filled points indicate treatment means, with vertical error bars showing SE.
**Figure S5:** Relationship between species richness and Blüthgen's d′ specialization index in the montane and the tundra grassland. Points represent mean values for each species (plants) and families (insects) coloured by treatments: Control (blue), Fence (orange), NPK (brown), and NPK + Fence (pink). Solid black lines indicate linear model fits, and shaded areas represent 95% confidence intervals.
**Figure S6:** Relationship between Shannon diversity index and Blüthgen's d′ specialization index in the montane and the tundra grassland. Points represent the mean value for each plot and treatment: Control (blue), Fence (orange), NPK (brown), and NPK + Fence (pink). Solid black lines indicate linear model fits, and shaded areas represent 95% confidence intervals.
**Table S5:** Full mixed effects model summary of log transformed family richness. Significant effects (*p* < 0.05) are shown in bold.
**Table S6:** Full mixed effects model summary of Shannon diversity. Significant effects (*p* < 0.05) are shown in bold.
**Table S7:** Full mixed effects model summary of the number of interactions. Significant effects (*p* < 0.05) are shown in bold.
**Table S8:** Full mixed effects model summary of weighted connectance. Significant effects (*p* < 0.05) are shown in bold.
**Table S9:** Full mixed effects model summaries of H_2_′ and nestedness of flower‐visitor networks in the montane grassland. Significant effects (*p* < 0.05) are shown in bold.
**Table S10:** Full mixed effects model summary of Blüthgen's d′ values of plants and insects in the montane grassland. Significant effects (*p* < 0.05) are shown in bold.

## Data Availability

The data and code supporting the findings of this study are publicly available in the Dryad repository at https://doi.org/10.5061/dryad.7d7wm389r.
