# Supplementary material for: Pollination Under Pressure: Grazing and Nutrient Enrichment Reshape Arctic Pollinator Communities and Interaction Networks
Source: Glob Chang Biol. 2026 Aug 8;32(8):e71039. doi: 10.1111/gcb.71039 (PMC13451957; doi:10.1111/gcb.71039)
Supplement: Supplementary file 1 [file GCB-32-e71039-s001.docx]

**Supplemental materials**

**Fig. S1.** An ordination diagram of flower-visiting insect community composition (family level) in the tundra (green) and montane grassland (blue). The diagram shows differences in community structure among based on a NMDS multivariate analysis of insect-visitor abundance data. Polygons enclose treatment groups, with centroids connected by lines. Labels indicate the dominant insect families.

**Table S1**. Frequency table showing the abundance of insect families collected at the montane grassland site across treatments: Control, Fence, NPK, and NPK + Fence. Each value represents the total number of individuals recorded for a given family within each treatment. Total sample sizes for each treatment are shown at the bottom of the plot, and overall family totals are shown to the right.

**Table S2**. Frequency table showing the abundance of insect families collected at the tundra grassland site across treatments: Control, Fence, NPK, and NPK + Fence. Each value represents the total number of individuals recorded for a given family within each treatment. Total sample sizes for each treatment are shown at the bottom of the plot, and overall family totals are shown to the right.

**Table S3**. Frequency table showing the abundance of plant species at the montane grassland site across treatments: Control, Fence, NPK, and NPK + Fence. Each value represents the total number of individuals recorded for a given species within each treatment. Total sample sizes for each treatment are shown at the bottom of the plot, and overall totals for each taxon are shown to the right.

**Table. S4**. Frequency table showing the abundance of plant species at the tundra grassland site across treatments: Control, Fence, NPK, and NPK + Fence. Each value represents the total number of individuals recorded for a given species within each treatment. Total sample sizes for each treatment are shown at the bottom of the plot, and overall totals for each taxon are shown to the right.

**Fig. S2 a).** Weighted flower-visitor networks in the montane grassland constructed for experimental treatments: Control (grazed) and Fence (grazing exclusion). The rectangles on the right side represent plant species and flower-visitor families on the left side.

**Fig. S2 b).** Weighted flower-visitor networks in the montane grassland constructed for experimental treatments: NPK (nutrient addition) and NPK + Fence (nutrient addition and grazing exclusion combined). The rectangles on the right side represent plant species and flower-visitor families on the left side.

**Fig. S3.** Weighted flower-visitor networks in the tundra grassland constructed for each experimental treatment: Control (grazed), Fence (grazing exclusion), NPK (nutrient addition) and Fenced NPK (combined nutrient addition and grazing exclusion). The rectangles on the right side represent plant species and flower-visitor families on the left side.

**Fig. S4**. Relationship between Blüthgen’s d´specialization index for insects (green) and plants (blue) in a) the montane grassland and b) the tundra grassland. Points represent mean values for each species (plants) or families (insects) under four experimental treatments: Control, Fence, NPK and NPK + Fence. Large, filled points indicate treatment means, with vertical error bars showing SE.

**Fig. S5.** Relationship between species richness and Blüthgen’s d´ specialization index in the montane and the tundra grassland. Points represent mean values for each species (plants) and families (insects) coloured by treatments: Control (blue), Fence (orange), NPK (brown), and NPK + Fence (pink). Solid black lines indicate linear model fits, and shaded areas represent 95% confidence intervals.

**Fig. S6.** Relationship between Shannon diversity index and Blüthgen’s d´ specialization index in the montane and the tundra grassland. Points represent the mean value for each plot and treatment: Control (blue), Fence (orange), NPK (brown), and NPK + Fence (pink). Solid black lines indicate linear model fits, and shaded areas represent 95% confidence intervals.

**EMMEANS CONTRASTS BASED ON MIXED EFFECTS MODELS**

Emmeans contrasts based on the mixed effects models. The p-value adjustments of contrasts are based on Tukey method. In all cases the degrees-of-freedom based on Kenward-Roger method (Lenth & Piaskowski 2025). Significant effects (p < 0.05) are shown in bold.

Family richness (log) in the montane grassland (emmeans based on the simplified model)

```
 contrast              estimate    SE df t.ratio p.value
 Fen0 NPK0 - Fen1 NPK0   -0.496 0.166 10  -2.983  0.0565
 Fen0 NPK0 - Fen0 NPK1   -0.340 0.166 10  -2.046  0.2348
 Fen0 NPK0 - Fen1 NPK1   -0.836 0.235 10  -3.556  0.0227
 Fen1 NPK0 - Fen0 NPK1    0.156 0.235 10   0.662  0.9089
 Fen1 NPK0 - Fen1 NPK1   -0.340 0.166 10  -2.046  0.2348
 Fen0 NPK1 - Fen1 NPK1   -0.496 0.166 10  -2.983  0.0565
```

Family richness in the tundra grassland (emmeans based on the simplified model)

```
contrast              estimate    SE df t.ratio p.value
 Fen0 NPK0 - Fen1 NPK0   -1.237 0.243 10  -5.083  0.0022
 Fen0 NPK0 - Fen0 NPK1   -0.577 0.243 10  -2.372  0.1460
 Fen0 NPK0 - Fen1 NPK1   -1.814 0.344 10  -5.271  0.0017
 Fen1 NPK0 - Fen0 NPK1    0.660 0.344 10   1.917  0.2808
 Fen1 NPK0 - Fen1 NPK1   -0.577 0.243 10  -2.372  0.1460
 Fen0 NPK1 - Fen1 NPK1   -1.237 0.243 10  -5.083  0.0022
```

Shannon diversity in the montane grassland (emmeans based on the simplified model)

```
contrast              estimate    SE df t.ratio p.value
 Fen0 NPK0 - Fen1 NPK0  -0.2060 0.127 10  -1.620  0.4108
 Fen0 NPK0 - Fen0 NPK1  -0.3028 0.127 10  -2.381  0.1440
 Fen0 NPK0 - Fen1 NPK1  -0.5088 0.180 10  -2.829  0.0720
 Fen1 NPK0 - Fen0 NPK1  -0.0967 0.180 10  -0.538  0.9477
 Fen1 NPK0 - Fen1 NPK1  -0.3028 0.127 10  -2.381  0.1440
 Fen0 NPK1 - Fen1 NPK1  -0.2060 0.127 10  -1.620  0.4108
```

Shannon diversity in the tundra grassland (emmeans based on the simplified model)

```
contrast              estimate    SE df t.ratio p.value
 Fen0 NPK0 - Fen1 NPK0   -0.729 0.247 10  -2.954  0.0591
 Fen0 NPK0 - Fen0 NPK1   -0.357 0.247 10  -1.448  0.5003
 Fen0 NPK0 - Fen1 NPK1   -1.086 0.349 10  -3.113  0.0459
 Fen1 NPK0 - Fen0 NPK1    0.371 0.349 10   1.065  0.7173
 Fen1 NPK0 - Fen1 NPK1   -0.357 0.247 10  -1.448  0.5003
 Fen0 NPK1 - Fen1 NPK1   -0.729 0.247 10  -2.954  0.0591
```

Number of interactions in the montane grassland (emmeans based on the simplified model)

```
contrast                  estimate   SE df t.ratio p.value
 NPK0 Fence0 - NPK1 Fence0    -36.9 17.5 10  -2.101  0.2172
 NPK0 Fence0 - NPK0 Fence1    -97.9 17.5 10  -5.578  0.0011
 NPK0 Fence0 - NPK1 Fence1   -134.8 24.8 10  -5.430  0.0014
 NPK1 Fence0 - NPK0 Fence1    -61.0 24.8 10  -2.458  0.1281
 NPK1 Fence0 - NPK1 Fence1    -97.9 17.5 10  -5.578  0.0011
 NPK0 Fence1 - NPK1 Fence1    -36.9 17.5 10  -2.101  0.2172
```

Number of interactions in the tundra grassland (emmeans based on the full model)

```
contrast                  estimate   SE df t.ratio p.value
 NPK0 Fence0 - NPK1 Fence0    -6.75 1.99  9  -3.393  0.0333
 NPK0 Fence0 - NPK0 Fence1   -16.00 1.99  9  -8.042  0.0001
 NPK0 Fence0 - NPK1 Fence1   -35.75 1.99  9 -17.969  <.0001
 NPK1 Fence0 - NPK0 Fence1    -9.25 1.99  9  -4.649  0.0054
 NPK1 Fence0 - NPK1 Fence1   -29.00 1.99  9 -14.576  <.0001
 NPK0 Fence1 - NPK1 Fence1   -19.75 1.99  9  -9.927  <.0001
```

Weighted connectance in the montane grassland (emmeans based on the simplified model)

```
contrast                  estimate     SE df t.ratio p.value
 NPK0 Fence0 - NPK1 Fence0  0.00185 0.0125 10   0.148  0.9988
 NPK0 Fence0 - NPK0 Fence1  0.03324 0.0125 10   2.657  0.0942
 NPK0 Fence0 - NPK1 Fence1  0.03510 0.0177 10   1.984  0.2563
 NPK1 Fence0 - NPK0 Fence1  0.03139 0.0177 10   1.774  0.3392
 NPK1 Fence0 - NPK1 Fence1  0.03324 0.0125 10   2.657  0.0942
 NPK0 Fence1 - NPK1 Fence1  0.00185 0.0125 10   0.148  0.9988
```

Weighted connectance in the tundra grassland (emmeans based on the simplified model)

```
contrast                  estimate     SE df t.ratio p.value
 NPK0 Fence0 - NPK1 Fence0   0.0780 0.0253 10   3.078  0.0485
 NPK0 Fence0 - NPK0 Fence1   0.1653 0.0253 10   6.528  0.0003
 NPK0 Fence0 - NPK1 Fence1   0.2433 0.0358 10   6.793  0.0002
 NPK1 Fence0 - NPK0 Fence1   0.0874 0.0358 10   2.439  0.1319
 NPK1 Fence0 - NPK1 Fence1   0.1653 0.0253 10   6.528  0.0003
 NPK0 Fence1 - NPK1 Fence1   0.0780 0.0253 10   3.078  0.0485
```

Blüthgen’s d’ values for plants in the montane grassland (emmeans based on the simplified model)

```
contrast                  estimate     SE   df t.ratio p.value
 NPK0 Fence0 - NPK1 Fence0  0.06852 0.0419 90.9   1.636  0.3640
 NPK0 Fence0 - NPK0 Fence1  0.00533 0.0425 90.9   0.125  0.9993
 NPK0 Fence0 - NPK1 Fence1  0.07385 0.0645 90.7   1.146  0.6623
 NPK1 Fence0 - NPK0 Fence1 -0.06319 0.0545 91.2  -1.160  0.6535
 NPK1 Fence0 - NPK1 Fence1  0.00533 0.0425 90.9   0.125  0.9993
 NPK0 Fence1 - NPK1 Fence1  0.06852 0.0419 90.9   1.636  0.3640
```

Blüthgen’s d’ values for insects in the montane grassland (emmeans based on the simplified model)

```
contrast                  estimate     SE  df t.ratio p.value
 NPK0 Fence0 - NPK1 Fence0   0.0786 0.0256 188   3.068  0.0131
 NPK0 Fence0 - NPK0 Fence1   0.0558 0.0262 183   2.129  0.1479
 NPK0 Fence0 - NPK1 Fence1   0.1344 0.0363 186   3.706  0.0016
 NPK1 Fence0 - NPK0 Fence1  -0.0228 0.0370 186  -0.614  0.9274
 NPK1 Fence0 - NPK1 Fence1   0.0558 0.0262 183   2.129  0.1479
 NPK0 Fence1 - NPK1 Fence1   0.0786 0.0256 188   3.068  0.0131
```

**Full mixed models**

**Table S5**. Full mixed effects model summary of log transformed family richness. Significant effects (p < 0.05) are shown in bold.

|  | **Montane grassland** | | | **Tundra grassland** | | |
| --- | --- | --- | --- | --- | --- | --- |
| Predictors | Estimates | CI | p | Estimates | CI | p |
| (Intercept) | 2.00 | 1.61 – 2.38 | **<0.001** | 0.45 | -0.15 – 1.05 | 0.126 |
| Fence | 0.45 | -0.09 – 1.00 | 0.093 | 1.24 | 0.43 – 2.04 | **0.007** |
| NPK | 0.30 | -0.25 – 0.84 | 0.250 | 0.58 | -0.23 – 1.38 | 0.144 |
| Fence × NPK | 0.08 | -0.69 – 0.85 | 0.813 | 0.00 | -1.14 – 1.15 | 0.995 |
| **Random Effects** | | | | | | |
| σ^2^ | 0.12 | | | 0.26 | | |
| τ_00_ | 0.00 _Blocks_ | | | 0.02 _Blocks_ | | |
| ICC |  | | | 0.09 | | |
| N | 4 _Blocks_ | | | 4 _Blocks_ | | |
| Observations | 16 | | | 16 | | |
| Marginal R^2^ / Conditional R^2^ | 0.448 / NA | | | 0.633 / 0.665 | | |

**Table S6**. Full mixed effects model summary of Shannon diversity. Significant effects (p < 0.05) are shown in bold.

|  | **Montane grassland** | | | **Tundra grassland** | | |
| --- | --- | --- | --- | --- | --- | --- |
| Predictors | Estimates | CI | p | Estimates | CI | p |
| (Intercept) | 0.79 | 0.50 – 1.07 | **<0.001** | 0.36 | -0.21 – 0.93 | 0.193 |
| Fence | 0.09 | -0.31 – 0.50 | 0.621 | 0.74 | -0.07 – 1.55 | 0.069 |
| NPK | 0.19 | -0.21 – 0.59 | 0.321 | 0.37 | -0.44 – 1.18 | 0.333 |
| Fence × NPK | 0.23 | -0.34 – 0.80 | 0.397 | -0.02 | -1.17 – 1.12 | 0.963 |
| **Random Effects** | | | | | | |
| σ^2^ | 0.07 | | | 0.26 | | |
| τ_00_ | 0.00 _Blocks_ | | | 0.00 _Blocks_ | | |
| N | 4 _Blocks_ | | | 4 _Blocks_ | | |
| Observations | 16 | | | 16 | | |
| Marginal R^2^ / Conditional R^2^ | 0.373 / NA | | | 0.400 / NA | | |

**Table S7**. Full mixed effects model summary of the number of interactions. Significant effects (p < 0.05) are shown in bold.

|  | **Montane grassland** | | | **Tundra grassland** | | |
| --- | --- | --- | --- | --- | --- | --- |
| Predictors | Estimates | CI | p | Estimates | CI | P |
| (Intercept) | 72.25 | 27.11 – 117.39 | **0.005** | 3.75 | -1.16 – 8.66 | 0.120 |
| NPK | 42.00 | -16.04 – 100.04 | 0.138 | 6.75 | 2.32 – 11.18 | **0.007** |
| Fence | 103.00 | 44.96 – 161.04 | **0.003** | 16.00 | 11.57 – 20.43 | **<0.001** |
| NPK × Fence | -10.25 | -92.32 – 71.82 | 0.786 | 13.00 | 6.73 – 19.27 | **0.001** |
| **Random Effects** | | | | | | |
| σ^2^ | 1356.84 | | | 7.92 | | |
| τ_00_ | 284.85 _Blocks_ | | | 11.54 _Blocks_ | | |
| ICC | 0.17 | | | 0.59 | | |
| N | 4 _Blocks_ | | | 4 _Blocks_ | | |
| Observations | 16 | | | 16 | | |
| Marginal R^2^ / Conditional R^2^ | 0.640 / 0.703 | | | 0.908 / 0.963 | | |

**Table S8**. Full mixed effects model summary of weighted connectance. Significant effects (p < 0.05) are shown in bold.

|  | **Montane grassland** | | | **Tundra grassland** | | |
| --- | --- | --- | --- | --- | --- | --- |
| Predictors | Estimates | CI | p | Estimates | CI | p |
| (Intercept) | 0.17 | 0.14 – 0.20 | **<0.001** | 0.47 | 0.41 – 0.53 | **<0.001** |
| NPK | -0.01 | -0.05 – 0.03 | 0.644 | -0.10 | -0.18 – -0.01 | **0.026** |
| Fence | -0.04 | -0.08 – 0.00 | 0.054 | -0.18 | -0.27 – -0.10 | **0.001** |
| NPK × Fence | 0.01 | -0.04 – 0.07 | 0.608 | 0.04 | -0.08 – 0.15 | 0.498 |
| **Random Effects** | | | | | | |
| σ^2^ | 0.00 | | | 0.00 | | |
| τ_00_ | 0.00 _Blocks_ | | | 0.00 _Blocks_ | | |
| ICC |  | | | 0.03 | | |
| N | 4 _Blocks_ | | | 4 _Blocks_ | | |
| Observations | 16 | | | 16 | | |
| Marginal R^2^ / Conditional R^2^ | 0.314 / NA | | | 0.764 / 0.770 | | |

**Table S9**. Full mixed effects model summaries of H_2_’ and nestedness of flower-visitor networks in the montane grassland. Significant effects (p < 0.05) are shown in bold.

|  | **H_2_’** | | | **Nestedness** | | |
| --- | --- | --- | --- | --- | --- | --- |
| Predictors | Estimates | CI | p | Estimates | CI | p |
| (Intercept) | 0.20 | 0.14 – 0.26 | **<0.001** | 14.35 | 7.29 – 21.42 | **0.001** |
| NPK | -0.07 | -0.15 – 0.02 | 0.125 | -1.88 | -11.87 – 8.11 | 0.684 |
| Fence | 0.03 | -0.05 – 0.12 | 0.403 | 6.87 | -3.12 – 16.86 | 0.156 |
| NPK × Fence | 0.01 | -0.12 – 0.13 | 0.912 | -1.41 | -15.54 – 12.72 | 0.828 |
| σ^2^ | 0.00 | | | 40.19 | | |
| τ_00_ | 0.00 _Blocks_ | | | 0.00 _Blocks_ | | |
| ICC |  | | |  | | |
| N | 4 _Blocks_ | | | 4 _Blocks_ | | |
| Observations | 16 | | | 16 | | |
| Marginal R^2^ / Conditional R^2^ | 0.315 / NA | | | 0.231 / NA | | |

**Table S10**. Full mixed effects model summary of Blüthgen’s d’ values of plants and insects in the montane grassland. Significant effects (p < 0.05) are shown in bold.

| **Montane grassland** | | | | | | |
| --- | --- | --- | --- | --- | --- | --- |
|  | **Plants** | | | **Insects** | | |
| *Predictors* | *Estimates* | *CI* | *p* | *Estimates* | *CI* | *p* |
| (Intercept) | 0.18 | 0.09 – 0.26 | **<0.001** | 0.22 | 0.16 – 0.28 | **<0.001** |
| NPK | -0.07 | -0.18 – 0.04 | 0.195 | -0.08 | -0.16 – 0.00 | 0.062 |
| Fence | -0.01 | -0.12 – 0.11 | 0.883 | -0.05 | -0.13 – 0.03 | 0.181 |
| NPK × Fence | 0.01 | -0.16 – 0.18 | 0.934 | -0.00 | -0.11 – 0.10 | 0.942 |
| **Random Effects** | | | | | | |
| σ^2^ | 0.04 | | | 0.03 | | |
| τ_00_ | 0.00 _block_ | | | 0.00 _block_ | | |
| N | 4 _block_ | | | 4 _block_ | | |
| Observations | 96 | | | 192 | | |
| Marginal R^2^ / Conditional R^2^ | 0.028 / NA | | | 0.071 / NA | | |
